# Three Rings of Polyhedral Simple Functions

**DOI:** 10.6028/jres.111.012

**Published:** 2006-04-01

**Authors:** Jim Lawrence

**Affiliations:** Department of Mathematical Sciences, George Mason University, Fairfax, VA 22030 and National Institute of Standards and Technology, Gaithersburg, MD 20899-8910

**Keywords:** convex polyhedra, Ehrhart polynomials, Gram’s relation, mixed volumes, transversal characteristic, valuation

## Abstract

We survey three ways to multiply elements of the additive subgroup of the group of real-valued functions on *R^d^* which is generated by the indicator functions of polyhedra. In the resulting commutative rings, identities often correspond to useful techniques of decomposition of polyhedra. We are led immediately to various interesting topics, including Ehrhart polynomials, mixed volumes, Gram’s relation, and transversal characteristics.

## 1. Introduction

Let $S^{d}$ denote the group of *polyhedral simple functions on R^d^*, the additive subgroup of the group of *Z*-valued functions on *R^d^* which is generated by the indicator functions of polyhedra. Here we study three ways to introduce multiplication on $S^{d}$, each giving it the structure of a commutative ring. The purpose of this paper is to note parallels among these three constructions, involving identities in the rings and resulting methods of decomposition of convex polyhedra. These considerations lead immediately to the principle of inclusion-exclusion, the combinatorial form of Gram’s relation, Ehrhart polynomials of polytopes, and other useful notions.

Each of the multiplications we consider arises from a binary operation on closed convex polyhedra; the three binary operations are intersection, convex hull of the union, and Minkowski addition.

For background on convex sets and polyhedra, consult the book by Stoer and Witzgall, [10].

## 2. The Principle of Inclusion-Exclusion

As the first example, illustrating the basic ideas, we describe a ring in which multiplication is related to intersection, and we use the ring structure to derive the principle of inclusion-exclusion.

If *P* ⊆ *R^d^* we let [*P*] denote its indicator function, [*P*]: *R^d^* → *Z*:

$$[P](x)=\{\begin{array}{cc} 1 & \text{if}\;x\in P,\\ 0 & \text{if}\;x\in P. \end{array}$$

The (additive) group of *simple functions* consists of the functions *F*: *R^d^* → *Z* of the form

$$F(x)=\sum_{i=1}^{n}\alpha_{i}[P_{i}](x),$$

where the *α_i_*’s are integers and the *P_i_*’s are sets in *R^d^*. These are the *Z*-valued functions having finitely many values. In later sections, we will be interested in the subgroup of the group of simple functions consisting of the *polyhedral* simple functions, the functions having such a representation, in which the *P_i_*’s are polyhedra. Here, a polyhedron is a finite union of sets which are relative interiors of convex polyhedra. Equivalently, it is an element of the Boolean lattice generated under intersection, union, and complementation by the closed halfspaces. The polyhedral simple functions are those functions *F*: *R^d^* → *Z* having finitely many values and such that, for each *k* ∈ *Z*, the inverse image of *k*, the set *F*^−1^(*k*) = {*x* ∈ *R^d^*: *F*(*x*) = *k*}, is a polyhedron. The group of polyhedral simple functions on *R^d^* is denoted by $S^{d}$.

In this section, the multiplication we describe is defined on the collection consisting of all the *Z*-valued simple functions on *R^d^*; $S^{d}$ forms a subring.

The operation under consideration in this section is intersection, ∩. Is there a multiplication, to be denoted by ·, on the group of simple functions which gives it the structure of a commutative ring, such that, whenever *P* and *Q* are sets in *R^d^*, we have

[P]⋅[Q]=[P∩Q]?

Of course, the answer is “Yes”; for simple functions *F*,*G*, we take

(F⋅G)(x)=F(x)G(x),

pointwise multiplication. For *F* = [*P*] and *G* = [*Q*], we have *F* · *G* = [*P* ∩ *Q*].

Furthermore, this multiplication is unique. If

$$F=\sum_{i}\alpha_{i}[P_{i}]\;\text{and}\;G=\sum_{j}\beta_{j}[Q_{j}],$$

then we must have

$$F\cdot G=\sum_{i,j}\alpha_{i}\beta_{j}[P_{i}\cap Q_{j}];$$

then *F* · *G* must be the function which, for *x* ∈ *R^d^*, maps

$$\begin{array}{c} x\mapsto \sum_{i,j}\alpha_{i}\beta_{j}[P_{i}\cap Q_{j}](x)\\ =\sum_{i,j}\alpha_{i}\beta_{j}[P_{i}](x)[Q_{j}](x)\\ =\left(\sum_{i}\alpha_{i}[P_{i}](x)\right)\left(\sum_{j}\beta_{j}[Q_{j}](x)\right)\\ =F(x)G(x). \end{array}$$

Now suppose *A*_1_, *A*_2_, and *B* are sets in *R^d^*, with *B* = *A*_1_ ∪ *A*_2_. Then

$$([B]-[A_{1}])\cdot ([B]-[A_{2}])=0,$$

as is clear, since, for any *x* ∈ *R^d^*, evaluating the left-hand side yields ([*B*](*x*) − [*A*_1_](*x*))([*B*](*x*) − [*A*_2_](*x*)), and since *B* = *A*_1_ ∪ *A*_2_, at least one of the integers [*B*](*x*) − [*A*_1_](*x*) and [*B*](*x*) − [*A*_2_](*x*) must be zero. (Here we use that the function on simple functions which takes *F* to *F*(*x*)—evaluation at *x*—is a homomorphism of the ring, and that the simple function *F* is zero if and only if, for each *x* ∈ *R^d^*, *F*(*x*) = 0.) Expanding, we get

$$\begin{array}{c} 0=[B]\cdot [B]-[B]\cdot [A_{1}]-[A_{2}]\cdot [B]+[A_{1}]\cdot [A_{2}]\\ =[B]-[A_{1}]-[A_{2}]+[A_{1}\cap A_{2}], \end{array}$$

or, for each *x* ∈ *R^d^*,

$$[B](x)=[A_{1}](x)+[A_{2}](x)-[A_{1}\cap A_{2}](x).$$

This yields the simple counting principle called the “principle of inclusion-exclusion” in the case of two sets, *A*_1_, *A*_2_: If *B* is finite then summing both sides of the last equation over all *x* ∈ *R^d^* (essentially a finite sum, since finiteness of *B* ensures that there are only finitely many *x* ∈ *R^d^* for which the values are nonzero), we obtain the cardinality of *B* in the equation

$$|B|=|A_{1}|+|A_{2}|-|A_{1}\cap A_{2}|.$$

Similar consequences hold when summing or integrating other functions over a set *B*.

More generally, for an arbitrary number *n* of sets *A_i_*, letting *B* = *A*_1_ ∪ ⋯ ∪ *A_n_* ⊆ *R^d^*, we have

$$([B]-[A_{1}])\cdot ([B]-[A_{2}])\cdot \ldots \cdot ([B]-[A_{n}])=0.$$

Expanding as before we get an expression for [*B*] in terms of the indicator functions of the intersections of the *A_i_*’s:

$$[B]=\sum_{\begin{array}{l} \Lambda \subseteq [n]\\ \Lambda \neq \emptyset \end{array}}{\left(-1\right)}^{\left|\Lambda \right|-1}\left[\underset{i\in \Lambda }{\cap }A_{i}\right].$$

## 3. Convex Hull and Gram’s Relation

In this section, we consider what happens when we substitute the binary operation of taking the convex hull of the union for that of intersection (of the preceding section). Does there exist a multiplication ∨ on the additive group of polyhedral simple functions, $S^{d}$, such that, for the indicator functions of any nonempty closed convex polyhedra *P* and *Q*, the product is given by the indicator of their convex hull,

[P]∨[Q]=[conv(P∪Q)]?

Indeed there is such a product.

In the preceding section it was not necessary to restrict the simple functions which were considered, to be in the group $S^{d}$ of polyhedral simple functions. In this section and the next, this restrictive assumption will be made. We note, however, that we could equally well make one of two somewhat less restrictive assumptions; we could assume that the sets *F*^−1^(*k*) lie either in the Boolean lattice generated by the collection of open convex sets, or in that generated by the collection of closed convex sets. The group of polyhedral simple functions is a subgroup of each of these larger groups. See [6].

There is a very useful homomorphism $\bar{\chi }:S^{d}\to Z$. We briefly describe how to obtain it, using the Euler characteristic.

The (topological) Euler characteristic *χ* is defined on polyhedra by the following properties:

1. *χ*(Ø) = 0;
2. If *P* is the relative interior of a nonempty convex polyhedron of dimension *k* then *χ*(*P*) = (−1)*^k^*;
3. If *P* is partitioned into polyhedra *P_i_* (1 ≤ *i* ≤ *m*), so that *P* = ∪*_i_ P_i_*, and, for *i* ≠ *j*, *P_i_* ∩ *P_j_* = Ø, then *χ*(*P*) = Σ*_i_χ*(*P_i_*).

Any polyhedron has an Euler characteristic, and if *P*, *Q* ∈ *R^d^* are homeomorphic polyhedra, then *χ*(*P*) = *χ*(*Q*).

If *P* ⊆ *R^d^* is a nonempty open convex polyhedron then *χ*(*P*) = (−1)*^d^*, while *χ*(Ø) = 0. These and the fact that *χ* has the additivity property, *χ*(*P* ∪ *Q*) + *χ*(*P* ∩ *Q*) = *χ*(*P*) + *χ*(*Q*) for any polyhedra *P*,*Q*, determine it uniquely.

See [7] for an easy proof of the existence of the function *χ* on polyhedra and its use in establishing Euler’s relation for the *f*-vector of a convex polytope. In this paper we have restricted the discussion to polyhedral simple functions; however, most of the results hold more generally for either the group of simple functions generated by the indicator functions of open convex sets, or that generated by indicator functions of closed convex sets. Two useful tools for accomplishing these extensions may be found in [4]; they are additive functions on the Boolean lattices, namely, the Euler characteristic for the family of open convex sets, which has value 1 on all nonempty open convex sets, and the Euler characteristic for the family of closed convex sets, which has value 1 on all nonempty closed convex sets (both functions having value 0 on the empty set). The first of these Euler characteristics, when restricted to polyhedra, yields the function *χ* above multiplied by a factor of (−1)*^d^*, while the second yields the function *χ*′ described below. These functions differ only by a multiplicative factor of (−1)*^d^* when applied to bounded polyhedra. See also [6], where the relationship between the two functions is made clear by making use of the “Sallee-Shephard mapping” which is introduced in that paper.

If *P* is a nonempty compact convex polyhedron—a nonempty convex polytope—then *χ*(*P*) = 1, while if *P* is a pointed unbounded closed convex polyhedron then *χ*(*P*) = 0. There is a second “Euler characteristic” *χ*′ characterized by the additivity property, *χ*′(Ø) = 0, and *χ*′(*P*) = 1 for any nonempty closed convex polyhedron *P*. For any polyhedron *P*, *χ*′(*P*) may be obtained by taking *χ*′(*P*) = *χ*(*P*) + *χ*(*P* ∩ ∂(*λC^d^*)), for sufficiently large *λ* ∈ *R*, where *C^d^* denotes the cube, *C^d^* = {*x* = (*x*_1_, …, *x_d_*) ∈ *R^d:^* −1 ≤ *x_i_* ≤ 1 for 1 ≤ *i* ≤ *d*} and ∂(*λC^d^*) represents the boundary of the dilation by a factor of *λ* of the cube. Here we dispense with *χ* in favor of *χ*′, as being better suited to the current needs. It has value 1 on every nonempty closed convex polyhedron, while *χ*′(Ø) = 0; and these properties together with additivity characterize it. However it is not invariant under homeomorphism.

Given $F\in S^{d}$ and *k* ∈ *Z*, the inverse image *F*^−1^(*k*) is a polyhedron, since $F\in S^{d}$; and it is empty for all but finitely many integers *k*. Therefore, we may define

$$\bar{\chi }(F)=\sum_{k\in Z}k\chi^{\prime }(F^{-1}(k)),$$

essentially a finite sum. Note that since the nonempty sets of the form *F*^−1^(*k*) partition *R^d^*,

$$\sum_{k\in Z}\chi^{\prime }\left(F^{-1}\left(k\right)\right)=\chi^{\prime }\left(R^{d}\right)=1.$$

**Lemma 1** *The function* $\bar{\chi }:S^{d}\to Z$ *is a group homomorphism. If P is a nonempty closed convex polyhedron then* $\bar{\chi }:([P])=1$.

*Proof.* That $\bar{\chi }:S^{d}\to Z$ is a group homomorphism is easily verified:

$$\begin{array}{c} \bar{\chi }(F+G)=\sum_{k}k\chi^{\prime }(\{x\in R^{d}:F(x)+G(x)=k\})\\ =\sum_{a,b}\left(a+b)\chi^{\prime }(F^{-1}(a)\cap G^{-1}(b)\right)\\ =\sum_{a}\sum_{b}a\chi^{\prime }(F^{-1}(a)\cap G^{-1}(b))\\ +\sum_{b}\sum_{a}b\chi^{\prime }(F^{-1}(a)\cap G^{-1}(b)), \end{array}$$

which, since the sets *F*^−1^(*a*) ∩ *G*^−1^(*b*), for fixed *a*, form a partition of *G*^−1^(*b*), and for fixed *b*, form a partition of *F*^−1^(*a*), may be continued,

$$\begin{array}{l} =\sum_{a}a\chi^{\prime }(F^{-1}(a))+\sum_{b}b\chi^{\prime }(G^{-1}(b))\\ =\bar{\chi }(F)+\bar{\chi }(G). \end{array}$$

If *P* is a closed convex polyhedron then, letting *F* denote [*P*], $\bar{\chi }(F)=\chi^{\prime }(P)=1$, by definition.

The collection of evaluation mappings, used in the preceding section (but perhaps “transparently, to the user”) have simple properties which made them useful there. For *x* ∈ *R^d^*, denote by *ε_x_* the mapping $\varepsilon_{x}:S^{d}\to Z$ which maps *F* ⟼ *F*(*x*), for $F\in S^{d}$. These functions are group homomorphisms; they are ring homomorphisms, when $S^{d}$ is considered as a ring under intersection multiplication. Also, if $F\in S^{d}$ is not the zero function, then there exists an evaluation homomorphism *ε_x_* such that *ε_x_*(*F*) ≠ 0. Equivalently, if *F*, $G\in S^{d}$ then *F* = *G* if and only if, for each *x* ∈ *R^d^*, *ε_x_*(*F*) = *ε_x_*(*G*). We say that the evaluation homomorphisms *distinguish elements* of $S^{d}$. (Some authors would say that the set of evaluation homomorphisms constitutes a *separating set of functions*.) Using $\bar{\chi }$, we construct another collection of homomorphisms which distinguish elements of $S^{d}$.

Given an open halfspace *H* ⊆ *R^d^*, define

$$\varphi_{H}(F)=\bar{\chi }([H]\cdot F)\text{for}\;F\in S^{d}.$$

If *P* is a nonempty closed convex polyhedron we have

$$\varphi_{H}([P])=\{\begin{array}{cc} 1 & \text{if}\;P\subseteq H\\ 0 & \text{otherwise}, \end{array}$$

using

$$\varphi_{H}([P])=\bar{\chi }([H\cap P])=\bar{\chi }([(H\cap \partial H)\cap P])=\bar{\chi }([(\partial H)\cap P]),$$

which is easy to compute, since the arguments of $\bar{\chi }$ in the last expression are indicator functions of closed convex polyhedra (possibly empty).

**Lemma 2** *The mappings φ_H_ are group homomorphisms. The collection of such mappings, together with* $\bar{\chi }$, *distinguish elements in* $S^{d}$. *If P, Q are nonempty closed convex polyhedra and H is an open halfspace, then*

$$\varphi_{H}([\text{conv}(P\cup Q)])=\varphi_{H}([P])\varphi_{H}([Q]).$$

*Proof*. The first and last sentences of the lemma are immediate from what has just been stated and the properties of $\bar{\chi }$. For the middle sentence, we refer the reader to Theorem 12 of [6], from which it can be easily derived.

In Theorem 1 we see that convex hull of the union yields a multiplicative structure on $S^{d}$.

**Theorem 1** *There is a unique multiplication* ∨ *on* $S^{d}$, *making* $S^{d}$ *into a (commutative) ring, such that, whenever P and Q are nonempty closed convex polyhedra*, [*P*] ∨ [*Q*] = [conv(*P* ∪ *Q*)]. *The mappings* $\varphi_{H}:S^{d}\to Z$ *and* $\bar{\chi }:S^{d}\to Z$ *are ring homomorphisms.*

*Proof*. As in the case of intersection, if such a multiplication exists, it is unique; for if

$$F=\sum_{i}\alpha_{i}[P_{i}]\;\text{and}\;G=\sum_{j}\beta_{j}[Q_{j}],$$

where the *P_i_*’s and *Q_j_*’s are nonempty closed convex polyhedra, then

$$\begin{array}{l} F\vee G=\sum_{i,j}\alpha_{i}\beta_{j}[P_{i}]\vee [Q_{j}]\\ =\sum_{i,j}\alpha_{i}\beta_{j}[\text{conv}(P_{i}\cup Q_{j})]. \end{array}$$

We use this as the definition for arbitrary *F*, $G\in S^{d}$ and show that it is well-defined. Denote the right-hand side in this equation by *J*. We have:

$$\begin{array}{c} \varphi_{H}(J)=\sum_{i,j}\alpha_{i}\beta_{j}\chi^{\prime }(H\cap \text{conv}(P_{i}\cup Q_{j}))\\ =\sum_{i,j}\alpha_{i}\beta_{j}\chi^{\prime }(H\cap P_{i})\chi^{\prime }(H\cap Q_{j})\\ =\sum_{i}\alpha_{i}\chi^{\prime }(H\cap P_{i})\sum_{j}\beta_{j}\chi^{\prime }(H\cap Q_{i}) \end{array}$$

This depends only on F and *G*, not on their representations, so, given different representations, resulting in *J*′ rather than *J* in the above, the value of *φ_H_*(*J*′) would be unchanged: *φ_H_*(*J*′) = *φ_H_*(*F*) *φ_H_*(*G*) = *φ_H_*(*J*). Replacing *φ_H_* by $\bar{\chi }$ in the above, we similarly get $\bar{\chi }(J^{\prime })=\bar{\chi }(J)$. From this and the fact that the homomorphisms *φ_H_*, $\bar{\chi }$ distinguish elements of $S^{d}$ it follows that *J*′ = *J*, so that *F* ∨ *G* is well-defined by the above expression.

We have seen that *φ_H_*(*F* ∨ *G*) = *φ_H_*(*F*) *φ_H_*(*G*), so *φ_H_* is a ring homomorphism. That $\bar{\chi }$ is also a ring homomorphism can be verified similarly without difficulty.

We use arithmetic in this ring to verify the combinatorial form of Gram’s relation, in Theorem 2, below.

**Lemma 3** *Let P* ⊆ *R^d^ be a nonempty convex polytope and let C*_1_, …, *C_n_ be the cones emanating from its vertices generated by P. Then*

$$([C_{1}]-[P])\vee ([C_{2}]-[P])\vee \cdots \vee ([C_{n}]-[P])=0.$$

*Proof*. Suppose *H* is an open halfspace. We consider the effect of applying *φ_H_* to the left-hand side. *P* ⊆ *H* if and only if there is a vertex of *P* for which the corresponding cone *C_j_* is contained in *H*. Therefore, if *P* ⊄ *H* then *φ_H_*([*C_i_*]) = *φ_H_*(*P*) = 0 for each *i*; and, otherwise, for this *j* we have *φ_H_*([*P*]) − *φ_H_*([*C_j_*]) = 0. In each case we have

$$\varphi_{H}(([C_{1}]-[P])\vee ([C_{2}]-[P])\vee \cdots \vee ([C_{n}]-[P]))=0.$$

This holds for each open halfspace *H*. Also, it is clear that $\bar{\chi }$, when applied to the left-hand side, yields 0. Since the mappings *φ_H_*, $\bar{\chi }$ distinguish elements of $S^{d}$, the stated equality follows.

Let *P* be as in the lemma, and suppose that the vertices of *P* are *v*_1_, …, *v_n_* and the cones at the vertices are, as before, *C*_1_, …, *C_n_*. For each nonempty face *F* of *P*, let *C_F_* denote the cone generated by *P* from *F*. It is easily verified that for Λ ⊆ [*n*], Λ ≠ Ø, conv(∪*_i_*_∈Λ_ *C_i_*) = *C_F_*, where *F* is the smallest face of *P* containing {*v_i_*: *i* ∈ Λ}.

**Theorem 2** (*Gram’s relation, combinatorial form*.)

$$[P]=\sum_{\begin{array}{l} F,\;\text{a face}\\ \text{of}\;P,F\neq \emptyset \end{array}}{\left(-1\right)}^{\dim(F)-1}[C_{F}].$$

*Proof*. Expanding the expression in the lemma leads immediately to the equation

$$[P]=\sum_{\begin{array}{l} \Lambda \subseteq [n],\\ \Lambda \neq \emptyset \end{array}}{\left(-1\right)}^{|\Lambda |-1}[\text{conv}(\underset{i\in \Lambda }{\cup }C_{i})].$$

For a nonempty face *F* of *P*, let Λ*_F_* denote the collection of subsets Λ ⊆ [*n*] such that the smallest face containing {*v_i:_ i* ∈ Λ} is *F*. The sets Λ*_F_*, for *F* a nonempty face, form a partition of the set of nonempty subsets of [*n*]. We then have

$$[P]=\sum_{\begin{array}{l} F,\;\text{a face}\\ \text{of}\;P,F\neq \emptyset \end{array}}\sum_{\Lambda \in \Lambda_{F}}{\left(-1\right)}^{|\Lambda |-1}[C_{F}].$$

The result now follows from the fact that

$$\sum_{\Lambda \in \Lambda_{F}}{\left(-1\right)}^{|\Lambda |-1}={\left(-1\right)}^{\dim(F)-1},$$

which can be derived as a consequence of Euler’s relation for the *f*-vectors of convex polytopes.

This theorem can be generalized in a way, by making use of the notion of the “transversal characteristic” of [6]. Given a nonempty finite collection $C=\{P_{1},\ldots ,P_{n}\}$ of closed convex polyhedra, the *transversal characteristic* of C is the element of $S^{d}$ given by

$$\tau [C]=\sum_{\begin{array}{l} \Lambda \subseteq [n],\\ \Lambda \neq \emptyset \end{array}}{\left(-1\right)}^{|\Lambda |-1}[\text{conv}(\underset{i\in \Lambda }{\cup }P_{i})].$$

A convex set *C* is a *transversal* of C if, for each *i* ∈ [n], *C* ∩ *P_i_* ≠ Ø.

**Theorem 3** *Two finite collections of closed convex polyhedra have the same convex transversals if and only if their transversal characteristics are equal.*

*Proof*. Let $C=\{P_{1},\ldots ,P_{n}\}$, as above. If, for some *i*, *P_i_* = Ø, then C has no convex transversals; and in this case it is easy to see that τ(C)=0. If no *P_i_* is empty then certainly convex transversals of C exist; also, in this case, $\bar{\chi }(\tau (C))=1$. Therefore the theorem holds whenever one of the two collections has Ø as an element. We may assume that this is not the case.

It is not difficult to verify that two collections have the same convex transversals if and only if the set of closed halfspaces which are transversals are the same for each.

Let *H* be an open halfspace. It is clear that

$$\varphi_{H}(\tau (C))=\sum_{\begin{array}{c} \Lambda \subseteq [n],\\ \Lambda \neq \emptyset \end{array}}{\left(-1\right)}^{|\Lambda |-1}\varphi_{H}([\text{conv}(\underset{i\in \Lambda }{\cup }P_{i})]).$$

Suppose then that *H* is an open halfspace whose complement is a transversal of C. In this case *H* contains none of the *P_i_*’s, so $\varphi_{H}(\tau (C))=0$. If, however, the complement of *H* is not a transversal of C, letting Γ = {*i* ∈ [*n*]: *P_i_* ⊆ *H*}, we have

$$\varphi_{H}([\text{conv}(\underset{i\in \Lambda }{\cup }P_{i})])=\{\begin{array}{cc} 1 & \text{if}\;\Lambda \subseteq \Gamma \\ 0 & \text{otherwise}. \end{array}$$

Then the summation reduces to

$$\varphi_{H}(\tau (C))=\sum_{\begin{array}{l} \Lambda \subseteq \Gamma ,\\ \Lambda \neq \emptyset \end{array}}{\left(-1\right)}^{|\Lambda |-1},$$

which has value 1, Γ being nonempty. Summarizing,

$$\varphi_{H}(\tau (C))=\{\begin{array}{ll} 0 & \text{if the complement of H is a transversal}\\ 1 & \text{if it is not}. \end{array}$$

It follows immediately that collections C and D of nonempty polytopes have the same transversals if and only if, for each open halfspace *H*, the values of *φ_H_* on their transversal characteristics are equal. Since also $\bar{\chi }(C)=\bar{\chi }(D)=1$, and the homomorphisms *φ_H_*, $\bar{\chi }$ distinguish elements of $S^{d}$, this happens if and only if their transversal characteristics are equal.

Gram’s relation is obtained from Theorem 3 by applying this theorem to the collection {*C*_1_, …, *C_n_*} of cones emanating from the vertices of *P*. This collection has the same convex transversals as {*P*}.

We call a family of convex sets *clustered* if each transversal is a transversal of the intersection of the family. In this case, as in Gram’s relation, the transversal characteristic is the indicator function of the intersection.

## 4. Minkowski Addition as the Product

Finally, we consider the existence of and a use for the multiplication *, for which, if *P* and *Q* are nonempty closed convex polyhedra in *R^d^*, [*P*] * [*Q*] = [*P* + *Q*]. This ring, studied by Groemer in [3], is called a “Minkowski ring” in [2] and [5]. The “polytope algebra” studied by McMullen in [8] and used by him to study the “*g*-theorem” for simplicial polytopes is a homomorphic image of a Minkowski ring.

It is necessary to introduce yet another collection of homomorphisms distinguishing elements of $S^{d}$. This time, the range will also be different. We denote by *T* the additive group of expressions which are finite sums of the form Σ*_i_α_i_t^ρi^*, where the *ρ_i_*’s are real numbers and the *α_i_*’s are integers. Elements of *T* may be viewed as functions on the nonnegative real numbers, and then *T* forms a ring, with pointwise multiplication as the product.

For each linear function *λ: R^d^* → *R*, we describe a homomorphism $\mu_{\lambda }:S^{d}\to T$. We define *µ_λ_* to be the (unique) homomorphism such that, for each nonempty closed convex polyhedron *P*,

$$\mu_{\lambda }([P_{i}])=\{\begin{array}{ll} t^{m} & \text{if}\;m=\max\{\lambda (x):x\in P\}\\ 0 & \text{otherwise}. \end{array}$$

**Lemma 4** *The homomorphism µ_λ_ is well-defined by the above. The homomorphisms of this form distinguish elements of* $S^{d}$.

*Proof*. Note that *Z* ⊆ *T* and if the linear functional *λ* is identically zero then *µ_λ_* is $\bar{\chi }:S^{d}\to Z\subseteq T$.

Suppose *λ* ≠ 0. Let *m* be a real number. For any real number *r*, let *H*(*r*) be the open halfspace *H*(*r*) = {*x* ∈ *R^d:^ λ*(*x*) < *r*}. For $F\in S^{d}$, consider

$$\alpha_{m}(F)=\underset{\varepsilon \to 0^{+}}{\lim}\varphi_{H(m+\varepsilon )}(F)-\varphi_{H(m)}(F).$$

It is easy to see that the limit exists, the function *φ_H_*_(_*_m_*_+_*_ε_*_)_(*F*) being constant for *ε* > 0 sufficiently small. Clearly $\alpha_{m}:S^{d}\to Z$ is a group homomorphism. Furthermore, if *P* is a closed convex polyhedron, then

$$\alpha_{m}([P])=\{\begin{array}{ll} 1 & \text{if}\;m=\max\{\lambda (x):x\in P\}\\ 0 & \text{otherwise}. \end{array}$$

It follows that *µ_λ_*(*F*) = Σ*_m_ α_m_*(*F*) *t^m^* for $F\in S^{d}$, a group homomorphism.

If *H* = {*x* ∈ *R^d^*: *λ*(*x*) < *r*}, we can retrieve *φ_Η_*(*F*) from *µ_λ_*(*F*): Litting $\mu_{\lambda }(F)=\sum_{m^{\prime }}\alpha_{m^{\prime }}t^{m^{\prime }}$, we have *φ_Η_*(*F*) = Σ{*α_m_*_′:_ *m*′ > *m*}. From this and the fact that the φ*_Η_*’s and $\bar{\chi }$ distinguish elements, it follows that the *µ_λ_*’s do so, as well.

In Theorem 4 we see that Minkowski addition yields a multiplicative structure on $S^{d}$.

**Theorem 4** There is a unique multiplication * on $S^{d}$ *such that, for closed convex polyhedra P and Q*, [*P*] * [*Q*] = [*P* + *Q*], *making* $S^{d}$ *into a commutative ring. The mappings* $\mu_{\lambda }:S^{d}\to T$ *and* $\bar{\chi }:S^{d}\to Z$ *are ring homomorphisms.*

*Proof*. The proof proceeds in a manner similar to that of Theorem 1. We skip the details, except to note that for nonempty closed convex polyhedra *P* and *Q* and a linear functional *λ*, *µ_λ_*([*P* + *Q*]) = *µ_λ_*([*P*])*µ_λ_*([*Q*]): *λ* is bounded above on *P* + *Q* if and only if it is bounded above on *P* and on *Q*, in which case, if *m_P_*_+_*_Q_*, *m_P_*, and *m_Q_* represent the three maximum values, we certainly have

Using the arithmetic of this ring will lead us quickly to the Ehrhart polynomials.

**Lemma 5** *Let P* ⊆ *R^d^ be a convex polytope having vertices v*_1_, *…, v_n_. Then, in the Minkowski ring*,

$$([P]-[\{v_{1}\}])*([P]-[\{v_{2}\}])*\cdots *([P]-[\{v_{n}\}])=0.$$

*Proof*. The proof proceeds in a manner similar to that of Lemma 3. This time we apply *µ_λ_* and note that the maximum of *λ* on a convex polytope occurs at a vertex.

In the following theorem it will be proven that a certain sequence of complex numbers, *s*_0_, *s*_1_, *s*_2_, …, is the sequence of values at the nonnegative integers of a polynomial. We briefly review the technique to be used to establish this. Suppose *s*_0_, *s*_1_, *s*_2_, …, is a sequence of complex numbers. We denote the sequence by the symbol s_*_. We form a new sequence Δs_*_, by taking differences of consecutive values: *s*_1_ − *s*_0_, *s*_2_ − *s*_1_, *s*_3_ − *s*_2_, …. It is true that s_*_ is the sequence of values of a polynomial if and only if Δs_*_ is the sequence of values of a polynomial; and, if s_*_ is not already the sequence of zeroes, then the degree of the polynomial representing Δs_*_ is one less than that representing s_*_ (taking the degree of the zero polynomial to be −1). Using this “difference” operator Δ multiple times, we see that s_*_ is the sequence of values of a polynomial of degree at most *n* − 1 if and only if Δ^n^s_*_ is the sequence of 0’s. We shall also make use of the fact that Δ^n^s_*_ is *t*_0_, *t*_1_, *t*_2_, …, where

$$t_{j}=\sum_{k=0}^{n}{\left(-1\right)}^{k}\left(\begin{array}{c} n\\ k \end{array}\right)S_{n-k+j}\;\text{for}\;j=0,1,2,\ldots .$$

**Theorem 5** *Let P* ⊆ *R^d^ be a convex polytope and let* P *denote the subring of* $S^{d}$ *generated by* [*P*] *and the elements* [{*x*}], *for x* ∈ *R^d^. Suppose γ is a homomorphism of the abelian groups which maps* P *to the additive group of complex numbers. Suppose that, for each vertex v of P and each F* ∈ *P, γ*(*F* * [*v*]) = *γ*(*F*). Then the sequence s_*_, *where sk= γ*([*P*] *^k^*), *is a polynomial of degree at most d.*

*Proof*. Note that *F* * [{*v*}] is the function taking *x* ⟼ *F*(*x* − *v*).

Expanding the left-hand side in the equation of Lemma 5 and multiplying by [*P*]*^j^*, we obtain

$$\sum_{\Lambda \subseteq [n]}{\left(-1\right)}^{\left|\Lambda \right|}\left(\prod_{i\in \Lambda }\left[\{v_{i}\}\right]\right){\left[P\right]}^{n-\left|\Lambda \right|+j}=0.$$

Here the products are of course multiplications in the Minkowski ring. Applying *γ* and using the fact that *γ*(*F*) = *γ*(*F* * [{*v_i_*}]) for any $F\in S^{d}$ and any *i*, we get

$$\sum_{\Lambda \subseteq [n]}{\left(-1\right)}^{\left|\Lambda \right|}\gamma ({\left[P\right]}^{n-\left|\Lambda \right|+j})=0,$$

which, upon counting sets, reduces to

$$\sum_{k=0}^{n}{\left(-1\right)}^{k}\left(\begin{array}{c} n\\ k \end{array}\right)\gamma ({\left[P\right]}^{n-k+j})=0;$$

then

$$\sum_{k=0}^{n}{\left(-1\right)}^{k}\left(\begin{array}{c} n\\ k \end{array}\right)S_{n-k+j}=0.$$

Comparing this with the expression preceding the statement of the theorem, we see that s_*_ is the sequence of values of a polynomial of degree at most *n* − 1.

We observe that the degree is actually no more than *d*. From a triangulation of *P* using simplexes having only vertices among those of *P*, [*P*] can be written as a sum-and-difference of indicator functions of simplexes. The sequence s_*_ of the theorem is then the sum-and-difference of those of the simplexes. Each of these is the sequence of values of a polynomial of degree at most *d*, so the same is true of s_*_.

Note that the group homomorphism *γ* need not be a ring homomorphism. It is easy to produce homomorphisms *γ* having the property required by the theorem. Here are two examples.

1. Let *Q* be a second convex polytope. For F∈P, let

$$\gamma (F)=\int_{R^{d}}[Q]*Fd\mu$$

where *µ* is ordinary Lebesgue measure.

1. Assume that the polytope *P* has only vertices in *Z^d^*. For F∈P, let

$$\gamma (F)=\sum_{z\in Z^{d}}F(z).$$

This is essentially a finite sum, since any element of P has bounded support.

In the first case, the terms of the sequence s_*_ are the volumes of the polytopes *Q* + *kP*, and the coefficients of the polynomial of the theorem, properly normalized, are the mixed volumes of *P* and *Q* ([3]); in the second, the terms are the cardinalities of the sets *Z^d^* ∩ *kP*, and the polynomial is the Ehrhart polynomial of *P*.

The Minkowski ring $S^{d}$ has a multiplicative identity element, [{0}], the function having value 1 at the origin and 0 elsewhere. The indicator functions of nonempty polytopes are invertible: [*P*]^−1^ = (−1)^dim(^*^P^*^)^[−*P*°], where −*P*° is the reflection through the origin of the relative interior *P*° of *P*. It is a simple extension of the theorem to show that the doubly infinite sequence s_*_ having *s_k_* = *γ*([*P*]*^k^*) for all *k* ∈ *Z* is a polynomial. Applied to Ehrhart polynomials, so that *P* and *γ* are as in the second case, we have that *s^k^* = |*Z^d^* ∩ *kP*| for *k* ≥ 0, and *s_k_* = (−1)^dim(^*^P^*^)^|*Z^d^* ∩ (−*k*)*P*°|, when *k* < 0. That the one polynomial serves both sequences is the reciprocity theorem studied by Ehrhart in [1], albeit in somewhat greater generality than is here described, and again by Stanley in [9].
